# Effects of High-Grain Diet With Buffering Agent on the Hepatic Metabolism in Lactating Goats

**DOI:** 10.3389/fphys.2019.00661

**Published:** 2019-05-29

**Authors:** Meilin He, Lin Li, Huanhuan Wang, Shuping Yan, Yuanshu Zhang

**Affiliations:** Key Laboratory of Animal Physiology and Biochemistry, Ministry of Agriculture, Nanjing Agricultural University, Nanjing, China

**Keywords:** comparative proteomic, buffering agent, liver, lactating goats, HPLC

## Abstract

To gain insight on the effects of a high-grain diet with buffering agent on liver metabolism and the changes of plasma biochemical parameters and amino acids in hepatic vein and portal vein, commercial kit and high performance liquid chromatography (HPLC) were applied to determine the concentration of amino acids of hepatic vein and portal vein blood samples, quantitative real-time PCR and comparative proteomic approach was employed to investigate proteins differentially expressed in liver in lactating dairy goats feeding high-grain diet with buffering agent or only high-grain diet. Results showed that feeding high-grain diet with buffering agent to lactating dairy goats could outstanding increase amino acid content of Gln (*p* < 0.01), and the amino acid contents of Arg and Tyr in BG were significantly higher (*p* < 0.05) than that in HG. After adding the buffering agent, the metabolism of amino acids in the liver were changed and most of the amino acids were increasingly synthesized and decreasingly consumed in the liver. In addition, 46 differentially expressed protein spots (≥1.5-fold changed) were detected in buffering group vs. control group using 2-DE technique and MALDI-TOF/TOF proteomics analyzer. Of these, 24 proteins showed increased expression and 22 proteins showed decreased expression in the buffer group vs. control group. Data on Gene Ontology (GO) analysis and Kyoto Encyclopedia of Genes and Genomes (KEGG) pathway analysis reveals that the high-grain diet with buffering agent alter the expression of proteins related to amino acids metabolism and glycometabolism. In addition, the results conclude that feeding high-grain diet with buffering agent can strengthen anti-oxidant capacity, stress ability, slow down urea metabolism, and alter amino acid metabolism as well as glycometabolism in the liver through different detection methods including proteomic analysis, real-time PCR analysis and biochemical analysis.

## Introduction

Due to degradation of grasslands coupled with shortage of greens fodders, per capita arable land has tremendously reduced and has left no option than to feed poor quality roughage. Consequently, there is a shift of feeding practices, the dairy industry uses high-grain diets to their herds.

Due to reduction of per capita arable land, the degradation of grasslands, and shortage of green fodder resources and poor quality, the current feeding practices in the dairy industry improving feed quality by feeding high-grain diets. However, many studies have confirmed that feeding of straw as main roughage with numerous high-grain diets improves the performance of ruminants but it can easily lead to subacute ruminal acidosis (SARA) (Hook et al., 2011; Dong et al., 2013). SARA has observed a decline in ruminal pH below 5.6 fed such ration for 3–5 h (AlZahal et al., 2007). In order to maintain ruminal pH in lactating dairy goats, buffering agent was added to the high-grain diets. For proper metabolic process the transportation of the nutrient from rumen to liver, nutrient must be transported from the rumen and gut to the liver (Annison and Bryden, 1998). The liver plays a crucial role in many essential metabolic processes of the body. Several enzymes in liver cells are required for key metabolic inter conversions. The effects of high-grain diets with buffering agent on liver function in ruminants are not well understood.

Proteomics is a powerful tool for the analysis of complex mixtures of proteins. At present, comparative proteomics provide a powerful approach in screening and alterations of protein levels (Dephoure and Gygi, 2012). Many studies have focused on characterizing diseased vs. normal liver proteomes (Parent and Beretta, 2005; Wang et al., 2012). The aim of this study was to determine changes in liver global protein expression in response to a high-grain diet with buffering agent; and to understand the effect of differentially expressed proteins for hepatic metabolism in lactating dairy goats. This study may help in uncovering the positive effects of feeding high-grain diet with buffering agent on hepatic metabolism in dairy goats, this study will postulate useful information for future studies in goat feeding.

## Materials and Methods

### Ethics Statement

All animal procedures were approved by the Institutional Animal Care and Use Committee of Nanjing Agricultural University. The protocols were reviewed and approved, and the project number 2011CB100802 was assigned. The slaughter and sampling procedures strictly followed the ‘*Guidelines on Ethical Treatment of Experimental Animals*’ (2006) no. 398 established by the Ministry of Science and Technology, China and the ‘*Regulation regarding the Management and Treatment of Experimental Animals*’ (2008) no. 45 set by the Jiangsu Provincial People’s Government.

### Experimental Animals

A total of 12 healthy multiparous mid-lactating goats (body weight, 38 ± 8 kg, mean ± SEM, 3–5 weeks post-partum) at the age of 2–3 years were used in experiments. They were housed in individual stalls in a standard animal feeding house at Nanjing Agricultural University (Nanjing, China). Goats were randomly divided into two groups, six in each group. Goats in two groups were fed the same experimental basic diet, and the mass ratio of refined feed and coarse feed were both 60:40, the ingredients and nutritional composition of the diets were presented in Table 1. NaHCO_3_ could increase the buffering capacity and prevent the acidosis in rumen. Diets with NaHCO_3_ did not result in as great a drop of rumen pH, and rumen pH was more stable for the post feeding (Snyder et al., 1983). Previous studies in our laboratory showed that feeding high-grain diet to dairy goats for a long-term would lead to the increase of rumen LPS in ruminants, which in turn resulted in the decrease of rumen pH. It is well documented that dietary addition of C_4_H_7_NaO_2_ could promote development of the rumen mucosa and health status of young dairy cows (Sander et al., 1959), and the addition of C_4_H_7_NaO_2_ to a high-grain diet could be given to lactating goats to decrease the content of LPS in rumen (Dai et al., 2017). Therefore, C_4_H_7_NaO_2_ and NaHCO_3_ were added in the buffering agent group on the basis of basic diet. The two groups were, respectively, high-grain diet group (Control, HG, concentrate: forage = 60:40) and buffering agent group (BG, concentrate: forage = 60:40 with 10 g C_4_H_7_NaO_2_ and 10 g NaHCO_3_), Dietary C_4_H_7_NaO_2_ and NaHCO_3_ were obtained from Nanjing Jiancheng Bioengineering Institute, China. The goats were fitted with a rumen fistula and hepatic catheters 2 weeks before the experiment and were ensured that they recovered from the surgery. Animals were monitored for 2 weeks after surgery. Sterilized heparin saline (500 IU/ml, 0.3 ml/time) was administered at 8-h intervals every day until the end of the experiment to prevent catheters from becoming blocked. During the experimental period of 20 weeks, goats were fed two times daily at 8.00 and 18.00, had free access to fresh water, and the feed amount met or exceeded the animal’s nutritional requirements. The Institutional Animal Care and Use Committee of Nanjing Agricultural University (Nanjing, People’s Republic of China) approved all of the procedures (surgical procedures and care of goats).

**Table 1:** Ingredients and nutritional composition of the diets.

Concentrate: Forage ratio 60:40		

Ingredient (%)		Nutrient levels^b^	
Leymus chinensis	27.00	Net energy/(MJ.kg^-1^)	6.71
Alfalfa silage	13.00	Crude protein/%	16.92
Corn	23.24	Neutral detergent fiber/%	31.45
Wheat bran	20.77	Acid detergent fiber/%	17.56
Soybean meal	13.67	Calcium/%	0.89
Limestone	1.42	Phosphorus/%	0.46
NaCl	0.30		
Premix^a^	0.60		
Total	100.00		


*^a^Provided per kg of diet: VA 6000 IU/kg, VD 2500 IU/kg, VE 80 mg/kg, Cu 6.25 mg/kg, Fe 62.5 mg/kg, Zn 62.5 mg/kg, Mn 50 mg/kg, I 0.125 mg/kg, Co 0.125 mg/kg.*

*^b^Nutrient levels were according to National Research Council (NRC, 2001).*

### Plasma Biochemical Parameters Analysis

At the 20th week, blood samples were collected from the hepatic vein and portal vein blood in 10 mL vacuum tubes containing sodium heparin. Blood was centrifuged at 3,000 ×*g* for 15 min to separate plasma, which was then stored at -20°C until analysis. After the blood plasma samples were obtained, they were sent to the hospital of integrated traditional Chinese and Western Medicine in Nanjing city to determine biochemical indicators.

### Analyses of HPLC

Free amino acids of hepatic vein and portal vein blood samples were determined by high performance liquid chromatography (HPLC), it was performed as previously described by Shen et al. (2010). The HPLC system consisted of: (1) Agilent1100 high-performance liquid chromatograph system (Agilent Technologies, Waldbronn, Germany); (2) scanning fluorescence detector (excitation 340 nm, emission 450 nm); (3) The chromatographic column (XTerra^®^MS C18, 4.6 mm × 250 mm, 5 μm), which was purchased from Waters (Waters Co., Milford, MA, United States). Twenty kinds of standard amino acids (Aldrich chemical company) were given by professor Holey, and the purity of these amino acids were greater than 98%. The three-dimensional flow phase (solution A, methanol; solution B, acetonitrile; solution C, 10 mmol/L phosphate buffer containing 0.3% tetrahydrofuran) was adopted. The gradient program was referred to Table 2, and the oven temperature was 40°C, the injection volume was 20 μL. The plasma samples were mixing with acetonitrile by 1:2 (v/v), and were placed at 4°C for 30 min, then they were centrifuged at 12,000 rpm for 30 min, and the supernatant fluids were collected for AA analysis. High pressure liquid chromatography analysis was performed after automatic pre-column derivatization with *O*-phthaldialdehyde (OPA) (Han et al., 2016).

**Table 2:** Gradient elution program of RP-HPLC.

Time (min)	Solution A (%)	Solution B (%)	Solution C (%)	Flow rate (mL/min)
0	85	6	9	1
10	80	8	12	1
25	70	15	15	1
40	45	25	30	1
50	45	25	30	1


### Sample Collection

After 20 weeks, all goats were killed via neck vein injections of xylazine [0.5 mg (kg body weight)-1; Xylosol; Ogris Pharme, Wels, Austria] and pentobarbital [50 mg (kg body weight)-1; Release; WDT, Garbsen, Germany]. After slaughter, liver tissue was collected and washed twice with cold physiological saline (0.9% NaCl) to remove blood. The livers were then transferred into liquid nitrogen and used for RNA and protein extraction.

### Two-Dimensional Gel Electrophoresis (2-DE)

The liver tissue samples of all goats in BG group in equal quality were mixed and washed three times with ice-cold saline containing 1 mM PMSF, then they were homogenized in the ice-cold lysis buffer [2 M thiourea, 7 M urea, 50 mM DTT, 2% (w/v) CHAPS, 0.5% (v/v) Bio-Lyte Ampholyte and 1 mM PMSF] by 1:5 (w/v). The homogenates were vortexed several times at maximum speed and kept at room temperature for 30 min, followed by centrifugation at 15,000 *g* for 30 min at 4°C. The protein content of the supernatant was determined using the Bradford assay. The HG group samples were treated in the same way. The total extraction protein was stored at -80°C for use. The first dimension used was isoelectric focusing (IEF). The extracted protein were diluted to 1000 mg/320 μL by rehydration loading buffer [2 Mthiourea, 7 M urea, 50 mM DTT, 2% (w/v) CHAPS, 0.5% (v/v) Bio-Lyte Ampholyte and 1 mM PMSF], and the diluted protein (320 μL) were loaded on a 17 cm IPG gel strip (non-linear, pH 3.0–10.0, Bio-Rad, United States). The isoelectric focusing process is referenced at Table 3. After IEF, the IPG strips were equilibrated by serial incubation for 15 min in equilibration buffer [6 M urea, 30% (v/v) glycerol, 2% (w/v) SDS, 50 mM Tris-HCl (pH 8.8) and 1% (w/v) DTT] and in equilibration buffer containing 2.5% (w/v) iodoacetamide instead of 1% DTT. Equilibrated IPG strips were transferred onto a 12.5% SDS-PAGE for the second dimension and covered them with 0.5% low melting point agarose containing 0.02% (w/v) bromophenol blue. Electrophoresis was run at bromophenol blue dye that reached the bottom to the edge of gels. Molecular masses were determined by precision protein standard markers (Bio-Rad), covering the range of 15–150 kDa. Gels were fixed in 12% trichloroacetic acid for 2 h, then stained with 0.08% (w/v) Coomassie Brilliant Blue G250 staining solution for 20 h. The excess of dye was removed with MilliQ water, and scanned with Molecular Imager (Versa Doc3000, Bio-Rad, United States) (Jiang et al., 2013). Standardization, background elimination, spot detection, gel matching and interclass analysis were performed as previously described using the PD Quest 8.0 software (Bio-Rad) (Yang et al., 2007). An average, three replicates were performed per sample. Protein spots were considered to be differentially expressed only if they showed 1.5-fold change in intensity, and satisfied the non-parametric Wilcoxon test (<0.05). Only the spots with the same changing trend in all three gels were considered for further analysis.

**Table 3:** Parameters for IEF of 2-DE (17 cm, pH 3.0–10.0 NL).

Step	Voltage	Step duration (h)/Vol-hours (V-h)	Ramp	Function
Rehydration	–	13 h	Passive hydration	Hydration
1	250 V	1 h	Chronic	Desalination
2	500 V	1 h	Chronic	Desalination
3	2,000 V	1 h	Linear	Desalination
4	8,000 V	3 h	Linear	boosting
5	8,000 V	60,000 V-h	Rapid	Focus
6	5,00 V	10 h	Rapid	Remain


### Trypsin Digestion and MS Analysis

Selected gel spots were manually excised and washed twice with MilliQ water. Trypsin digestion test was performed as described by Ura et al. (2016). The digested proteins were air-dried and analyzed by using a 4800 MALDI-TOF/TOF proteomics analyzer (Applied Biosystems, United States). A protein spot digested with trypsin was used to calibrate the mass spectrometer. A mass range of 800–3,500 Da was used. Combined search (MS plus MS/MS) was performed using GPS Explorer TM software v3.6 (Applied Biosystems, United States) and the MASCOT search engine (Matrix Science Ltd., United Kingdom). Proteins were considered as positive hints if at least two independent peptides were identified with medium (95%) or high (99%) confidence.

### Quantitative Real-Time PCR (qRT-PCR)

Relative mRNA expression in liver tissue was measured by qRT-PCR using the 2^-ΔΔCt^ method. Briefly, total RNA was extracted from liver samples using TRIzol reagent (Invitrogen, United States) and converted to cDNA using commercial kits (Vazyme, Nanjing, China). All PCR primers were synthesized by Generay Company (Shanghai, China), and the primer sequences are listed in Table 4. PCR was performed using the AceQ qPCR SYBR Green Master Mix kit (Vazyme, Nanjing, China) and the MyiQ2 Real-time PCR system (Bio-Rad, United States) with the following cycling conditions: 95°C for 2 min, 40 cycles of 95°C for 15 s and 60°C for 30 s. Glyceraldehyde 3-phosphate dehydrogenase (GAPDH) served as reference for normalization.

**Table 4:** The primer sequences and product sizes.

Gene name	Primer Sequence (5′→3′) Sense/Antisense	Product Size (bp)	Accession no.
Superoxide dismutase 1 (SOD1)	CATCCACTTCGAGGCAAAGG/TTGTCAGCCTTCACATTGCC	216	NM_001285550
Glutathione *S*-transferase A1(GSTA1)	CCAAGCTGACCCTAATCCGA/GAGGGAAGTTGGCCAAAAGG	183	BC102540
Catalase (CAT)	ACACAGGCACATGAACGGAT/GGCCGTAGTCAGGATCTTCG	161	XM_004016396
Glutathione peroxidase 1(GPX1)	CAGGAAAACGCCAAGAACGA/AAGTTCCAGGAGACGTCGTT	242	XM_004018462
Heat shock protein (HSP70)	GATCAACGACGGAGACAAGC/GCTGCGAGTCGTTGAAGTAG	182	JN604434
Regucalcin (RGN)	CCCTCTTTCCTGACCACCAT/TTCCCGTCTGCAGGTCATAG	150	EU797609
Protein disulfide isomerase family A member 3 (PDIA3)	ACAGAGTGATGATGGTGGCA/CGGTTCTGACAGCAACAACA	150	NM_001163045
Argininosucclnate synthetase (ASS)	TTCAAGGGCCAGGTGTACAT/GTCAGCTGGTCTATTTGGCG	190	M26198
Carbamyl phosphate synthetase I (CPS-I)	GCAATCATTCCGGCCAAGAT/TCACTAGGTCAATGCTGCCA	206	Y15793
Glyceraldehyde-3-phosphate dehydrogenase (GAPDH)	GGGTCATCATCTCTGCACCT/GGTCATAAGTCCCTCCACGA	180	HM043737


### Analysis of MDA Content and Total Anti-oxidative Capacity

The MDA content (catalog no. A0003-2, Jiancheng, Nanjing, China) and total anti-oxidative capacity of liver (catalog no. A015, Jiancheng, Nanjing, China) were analyzed by commercial kits. The procedures were performed according to the manufacturer’s instructions.

### Gene Ontology Analysis and KEGG Pathway Enrichment Analysis

The identified proteins were divided into groups based on the PANTHER classification system and using the enrichment analysis^1^, according to their biological processes, molecular function and cellular composition. This data could provide an overview of the main biological processes in which differentially expressed proteins participate. In addition, we also performed pathway enrichment analysis using the Kyoto Encyclopedia of Genes and Genomes (KEGG) pathway maps.

## Results

### The Plasma Biochemical Indexes

After adding buffering agent, the activities of ALT, AST, and LDH in hepatic vein plasma were decreased compared with BG, and these enzymes were associated with liver injury. It can be found that the consumption of glucose and total protein were reduced, and the synthesis of those were increased in buffering agent group by comparing the hepatic vein with portal vein plasma (Table 5).

**Table 5:** The plasma biochemical index (*n* = 6).

Index	Hepatic vein		Portal vein		Hepatic vein – Portal vein	
	HG	BG	HG	BG	HG	BG
GLU (mmol/L)	2.51 ± 0.08	3.26 ± 0.21^b^	3.04 ± 0.15	3.23 ± 0.37	–0.53 ± 0.08	0.03 ± 0.13
UR (mmol/L)	5.68 ± 0.37	5.19 ± 0.39	5.15 ± 0.39	4.85 ± 0.45	0.53 ± 0.11	0.35 ± 0.13
UA (μmol/L)	14.00 ± 0.41	13.50 ± 0.29	13.00 ± 0.41	12.75 ± 0.48	1.00 ± 0.71	0.75 ± 0.25
CRE (μmol/L)	40.50 ± 1.19	41.75 ± 1.11	39.25 ± 2.10	40.25 ± 1.31	1.25 ± 2.39	1.50 ± 2.36
BIL (μmol/L)	2.68 ± 0.25	2.75 ± 0.17^b^	2.42 ± 0.36	2.75 ± 0.18	0.47 ± 0.39	0.09 ± 0.08
ALT (IU/L)	24.50 ± 1.71	19.00 ± 1.08^b^	18.75 ± 0.48	16.50 ± 0.87	5.75 ± 2.06	2.50 ± 1.26
AST (IU/L)	153.00 ± 37.02	91.50 ± 21.67	77.00 ± 16.72	52.25 ± 5.19	76.00 ± 24.23	39.25 ± 16.70
AKP (IU/L)	45.25 ± 7.61	56.90 ± 8.60	32.50 ± 9.00	39.25 ± 9.69	12.75 ± 3.79	17.50 ± 5.56
CK (IU/L)	149.75 ± 10.32	127.70 ± 10.70	88.00 ± 4.40	72.00 ± 6.49	61.75 ± 8.96	55.00 ± 14.62
LDH (IU/L)	217.75 ± 1.65	206.50 ± 2.10^a^	176.50 ± 4.66	177.00 ± 4.60	41.25 ± 5.76	29.50 ± 6.13
TP (g/L)	54.20 ± 2.96	55.50 ± 3.13	62.25 ± 1.45	53.83 ± 3.55	–5.63 ± 3.76	1.68 ± 1.01
ALB (g/L)	24.35 ± 2.74	25.65 ± 1.92	29.55 ± 0.45	26.03 ± 2.59	–5.20 ± 2.87	–0.38 ± 0.87
GLO (g/L)	29.85 ± 0.69	29.85 ± 1.23	32.7 ± 1.30	27.8 ± 1.14^b^	–2.85 ± 1.11	2.05 ± 0.40


*GLU, glucose; UR, urea; UA, uric acid; CRE, creatinine; BIL, bilirubin; ALT, alanine transaminase; AST, aspartate aminotransferase; AKP, alkaline phosphatase; CK, creatine kinase; LDH, lactic dehydrogenase; TP, total protein; ALB, albumin; GLO, globulin. ^*a*^p < 0.01, ^*b*^p < 0.05 and compared with HG. n = 6/group.*

### Amino Acid Content in Plasma

**FIGURE 1:**
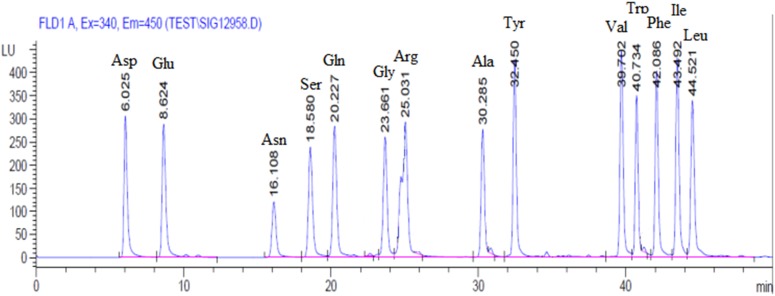
Representative chromatograms of amino acid profiles in plasma.

A chromatogram of synthetic mixture of amino acid standards was shown in Figure 1. Each peak represents one of specific amino acid, and 14 amino acids were separated (*T* < 50 min) under the experimental conditions used. In 31.25–500 μmol/L concentration range, amino acid standard concentration was linear related to the peak area and the correlation coefficients is 0.9960–0.9999. The Intra-day RSD and Inter-day RSD is between 2.15–4.05% and 3.13–4.83%, respectively, which are within 6%. These parameters results indicated that this sensitive procedure could be used for the quantitative analysis of amino acid in tissues.

As shown in Table 6, the amino acid content of Gln showed very significant difference (*p* < 0.01) between these two groups in hepatic venous blood. And the amino acid contents of Arg and Tyr in BG were significantly higher (*p* < 0.05) than that in HG. However, the difference of free amino acids in these two groups in the venous blood was not significant (*p* > 0.05). The comparison of free amino acid content in hepatic vein and venous blood showed that after adding the buffer agent, the metabolism of amino acids in the liver was changed and most of the amino acids were increasingly synthesized and decreasingly consumed in the liver.

**Table 6:** The plasma biochemical index (*n* = 6).

Free amino acid	Hepatic vein		Portal vein		Hepatic vein – Portal vein	
	HG	BG	HG	BG	HG	BG
Glu	241.87 ± 20.95	265.98 ± 12.38	363.25 ± 38.54	329.70 ± 40.13	–121.45 ± 53.41	–38.68 ± 47.91
Asn	607.58 ± 34.16	593.45 ± 52.21	338.30 ± 33.50	281.18 ± 36.65	269.32 ± 65.44	312.30 ± 81.18
Ser	766.80 ± 51.77	705.33 ± 64.90	308.15 ± 40.69	268.05 ± 38.27	458.65 ± 59.15	518.63 ± 85.15
Gln	263.53 ± 16.95	576.68 ± 46.53^a^	295.15 ± 44.84	219.68 ± 58.16	–31.58 ± 54.90	359.98 ± 55.49
Gly	1367.28 ± 118.35	1453.70 ± 172.21	1094.20 ± 141.93	921.10 ± 91.04	447.30 ± 124.24	721.83 ± 104.97
Arg	1234.18 ± 43.24	1444.63 ± 52.65^b^	1040.93 ± 56.54	1139.35 ± 36.70	193.20 ± 72.69	259.15 ± 79.87
Ala	710.65 ± 41.49	763.58 ± 51.58	768.43 ± 44.32	641.50 ± 18.80^b^	–57.78 ± 2.83	122.08 ± 63.60
Tyr	232.63 ± 10.85	313.70 ± 31.45^b^	261.13 ± 33.67	301.70 ± 3.88	–1.97 ± 21.52	37.00 ± 13.55
Val	1492.48 ± 24.35	1449.67 ± 21.46	1573.60 ± 52.99	1485.68 ± 48.11	–225.15 ± 111.55	–257.77 ± 141.00
Trp	272.23 ± 29.45	300.93 ± 20.50	265.13 ± 6.88	265.95 ± 7.31	62.87 ± 69.80	14.95 ± 49.42
Phe	157.70 ± 19.59	280.28 ± 49.42	165.23 ± 9.78	187.73 ± 42.61	–7.50 ± 27.09	67.53 ± 40.95
Ile	285.60 ± 28.01	305.93 ± 38.61	294.83 ± 14.39	298.98 ± 12.75	–106.63 ± 118.35	–60.50 ± 99.00
Leu	637.18 ± 28.01	715.13 ± 42.57	606.93 ± 28.18	655.25 ± 44.05	30.23 ± 58.46	59.93 ± 12.09


*^*a*^p < 0.01, ^*b*^p < 0.05 and compared with HG. n = 6/group.*

### Proteomic Studies

**FIGURE 2:**
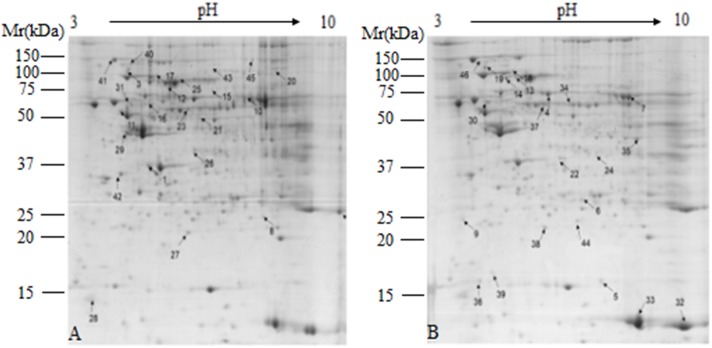
Representative 2-DE images of proteins extracted from lactating goat liver. **(A)** Control group; **(B)** buffer group. Equal amounts of protein (1,000 mg) were loaded onto the 17 cm IPG gel strip (non-linear, pH 3.0–10.0), and separated on 17-cm IPG strips, followed by electrophoresis on 12.5% SDS-PAGE gels for second dimension electrophoresis. Black arrows indicate differential protein spots (≥1.5-fold).

Comparative proteomic analysis was performed between HG and BG in lactating Saanen goats’ liver tissues, in order to understand the influence of adding buffering agent in High-Grain Diet on the hepatic metabolism. As shown at Figure 2, an average of 1,500 spots were detected on gels for both types of proteomes. We successfully identified a total of 46 differential protein spots (*p* < 0.05; in terms of expression, all 46 with a fold change ≥ 1.5-fold) that were differentially expressed in BG vs. HG using 2-DE technique and MALDI-TOF/TOF proteomics analyzer. Of these, 24 proteins showed increased expression and 22 proteins showed decreased expression in BG vs. HG, respectively. We can observe the main differential protein spots related to oxidative stress, amino acid metabolic process, Glycometabolism, TCA metabolic process and urea cycle depicted in Table 7. About twenty-two percent differential protein spots (10/46) were participated in oxidative stress. There were three differential protein spots involved in amino acid metabolic process, one differential protein spots were involved in urea cycle and two protein spots were involved in TCA metabolic process, glycometabolism, shown in two differential protein spots. The protein expression of most antioxidative differential protein spots were up-regulated in BG as compared to the HG, such as regucalcin (RGN, spot 1), protein disulfide isomerase A4 (PDIA4, spot 3), protein disulfide isomerase A3 (PDIA3, spot 4), peroxiredoxin-5 (PRDX5, spot 5), catalase (CAT, spot7) and aldehyde dehydrogenase, mitochondrial (ALDH2, spot16), and the protein expression of heat shock proteins (heat shock protein 70, HSPA1A, spot 12; heat shock cognate 71 kDa protein, HSPA8, spot 13; heat shock 70 kDa protein 6 isoform X2, HSPA6, spot 14) were also up-regulated, all of which are playing important roles in stress. The spots of malate dehydrogenase (MDH1, spot 19) and aconitate hydratase (ACO2, spot 20) participated in TCA metabolic process were down-regulated. In addition, the spots of carbamoyl phosphate synthase (CPS1, spot 22) related to urea cycle was down-regulated.

**Table 7:** Part of differential expression protein spots by MAIDI-TOF-TOF.

Spot no.	Identified protein name	Gene symbol	Accession no.	Mr (kDa)	pI	Protein expression	^1.5^Fold change
**Oxidative stress (10)**		
1	Regucalcin	RGN	gi| 193245514	34.0	5.42	Up	1.66
2	Glutathione *S*-transferase	GSTA1	gi| 5524944	25.3	8.67	Down	0.62
3	Protein disulfide isomerase A4	PDIA4	gi| 803277851	72.8	4.92	Up	2.94
4	Protein disulfide isomerase A3	PDIA3	gi| 239924054	57.4	6.23	Up	1.64
5	Peroxiredoxin-5, mitochondrial	PRDX5	gi| 426251990	23.4	8.62	Up	1.88
7	Catalase	CAT	gi| 426245288	60.2	6.77	Up	4.00
12	Heat shock protein 70	HSPA1A	gi| 371767262	70.5	5.82	Up	1.9
13	Heat shock cognate 71 kDa protein isoform X1	HSPA8	gi| 803156399	71.5	5.43	Up	1.9
14	Heat shock 70 kDa protein 6 isoform X2	HSPA6	gi| 803303190	71.4	5.74	Up	1.9
16	Aldehyde dehydrogenase, mitochondrial	ALDH2	gi| 426247368	57.1	7.55	Up	1.96
**Amino acid metabolic process (3)**		
10	Glutamate dehydrogenase	GLUD	gi| 21314225	16.9	9.30	Down	0.25
23	Cytosol aminopeptidase	LAP3	gi| 803082030	56.5	6.07	Up	1.96
27	Ornithine aminotransferase, mitochondrial isoform X2	OAT	gi| 803207295	37.8	8.37	Down	0.25
**Urea cycle (1)**		
22	Carbamoyl-phosphate synthase, mitochondrial isoform X1	CPS1	gi| 803028077	165.8	6.33	Down	0.27
**TCA metabolic process (2)**		
19	Malate dehydrogenase, cytoplasmic	MDH1	gi| 426223462	36.7	5.92	Down	0.54
20	Aconitate hydratase, mitochondrial isoform X1	ACO2	gi| 803058827	96.0	6.78	Down	0.07
**Glycometabolism (2)**		
21	Alpha-enolase isoform X3	ENO1	gi| 426239774	48.57	6.44	Up	1.55
35	Sorbitol dehydrogenase	SORD	gi| 330689592	38.5	7.27	Down	0.45


### Real-Time PCR Analysis

**FIGURE 3:**
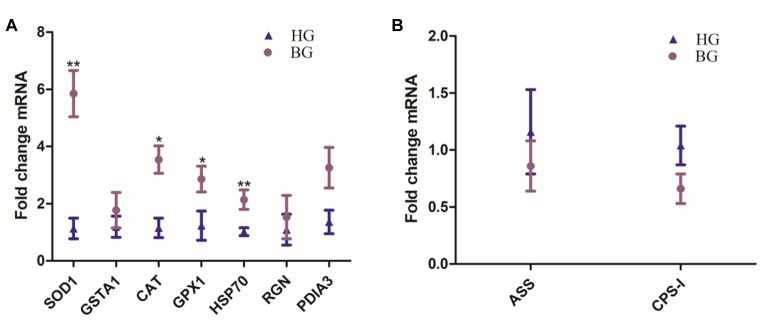
Effects of the BG diet on the expression of liver mRNA in lactating goats. **(A)** Hepatic SOD1, GSTA1, CAT, GPX1, HSP70, RGN, and PDIA3 mRNA expressions. **(B)** Hepatic ASS and CPS-I mRNA expressions. Values are mean ± SEM. ^∗∗^*P* ≤ 0.01, ^∗^*P* ≤ 0.05, BG vs. HG. *n* = 6/group.

Hepatic SOD1, GSTA1, CAT, GPX1, HSP70, RGN, and PDIA3 mRNA expressions were up-regulated in BG goats compared to HG goats, the mRNA expressions of CAT and GPX1 were significantly increased (*P* ≤ 0.05), and the mRNA expressions of SOD1 and HSP70 were very significantly increased (*P* ≤ 0.01) in BG goats compared to HG goats (Figure 3). ASS and -I were the limited enzyme in Urea synthesis and metabolism process, their mRNA expression levels were down-regulated but not prominently.

### Analysis of Malondialdehyde Content and Total Anti-oxidative Capacity

**FIGURE 4:**
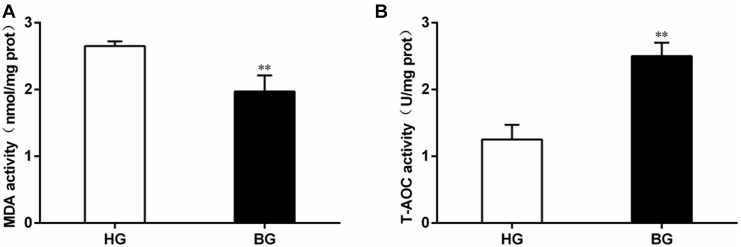
Analysis of MDA content and total anti-oxidative capacity. **(A)** The level of malondialdehyde (MDA) content; **(B)** the total anti-oxidative capacity (T-AOC). Values are mean ± SEM. ^∗∗^*P* ≤ 0.01, ^∗^*P* ≤ 0.05, BG vs. HG. *n* = 6/group.

The level of malondialdehyde (MDA) content was significantly decreased (*P* ≤ 0.05), but the total anti-oxidative capacity (T-AOC) was very significantly increased in liver of BG compared to HG goats (*P* ≤ 0.01) (Figure 4).

### GO Analysis of Differentially Expressed Proteins

**FIGURE 5:**
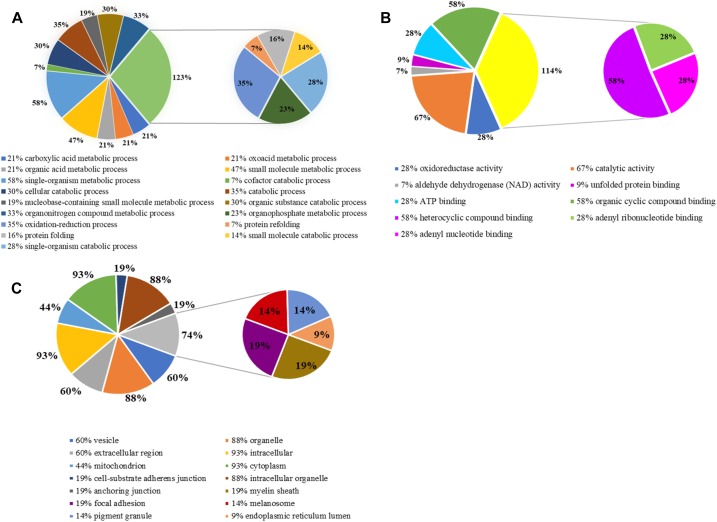
Gene ontology (GO) analysis of differentially expressed proteins. **(A)** Biological process; **(B)** molecular function; **(C)** cellular component.

Gene ontology is widely used to describe protein function in a standardized format. The gene symbols of 46 identified proteins were uploaded to the enrichment analysis of PANTHER classification system, three of which were unclassified and the rest of which (43) were included in our GO analysis (Figure 5). Three sets of ontology were classified. In terms of biological processes, the proteins were grouped into 17 main categories, 30% (13) of identified proteins were involved in cellular metabolic process, 35% (15) in oxidation-reduction process and catabolic process, 16% (7) in protein folding and 7% (3) in protein refolding; in terms of molecular function, 67% (29) in catalytic activity, 28% (12) in oxidoreductase activity; in terms of cellular component, 93% (40) proteins were related to cytoplasm, 88% (38) to intracellular organelle and 44% (19) proteins those play roles in mitochondrial metabolism.

### KEGG Pathway Analysis of Differentially Expressed Proteins

**FIGURE 6:**
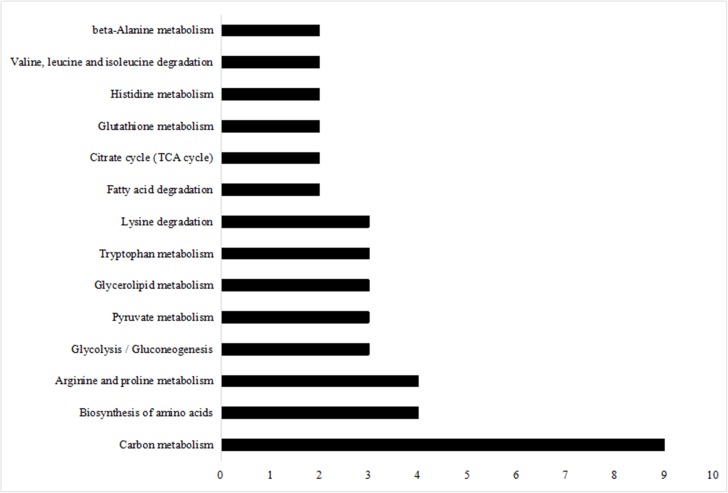
KEGG pathway analysis of differentially expressed proteins.

Kyoto Encyclopedia of Genes and Genomes pathway enrichment analysis is considered as one of the most reliable methods for functional annotation. We performed KEGG pathway enrichment analysis on the same set of differentially expressed proteins used for the GO analysis. As shown in Figure 6, the differentially expressed proteins were mainly involved in carbon metabolism, with a subset that was involved in biosynthesis of amino acids and arginine and proline metabolism.

## Discussion

In the present study, we used proteomic analysis on goat liver samples to identify differences in protein expression in BG vs. HG. Our proteomic data revealed that many interesting proteins were differentially expressed in the two groups. Prior studies have shown that feeding a high grain diet to lactating dairy cows could cause inflammatory response in liver of ruminants. Duanmu et al. (2016) have confirmed that high-grain diet causes acute phase response and oxidative stress. They found that differentially expressed proteins were involved in regulating oxidative stress in liver of lactating goats. Similarly in this study, we also found both of the oxidation resistance and stress ability were enhanced through different detection methods such as proteomic analysis, real-time PCR analysis and biochemical analysis. In addition, there were change in urea cycle, amino acid metabolism and glycometabolism in BG goats fed high-grain diet added with buffering agent as compared to HG group goats fed only high-grain diet.

### High-Grain Diet With Buffering Agent Strengthens Anti-oxidant Capacities

In this study, our proteomic data revealed that many interesting proteins were differentially expressed in the two groups. For example, regucalcin (RGN, spot 1) is a calcium-binding protein and modulates Ca^2+^ signaling. It is a regulatory protein in cell signaling system, which is considered to play a pivotal role in maintaining cell homeostasis and function (Yamaguchi, 2013). RGN has been found to stimulate SOD expression and help hepatic cell cytoplasm function in rats (Ichikawa and Yamaguchi, 2004). Down-regulation of RGN reduces SOD activity and thereby dysfunctions the antioxidant defense system (Fischer et al., 2012). SOD is an important antioxidant enzyme that can decrease the destruction of the superoxide radical by catalyzing dismutation and H_2_O_2_ formation (Kalyanaraman, 2013). Here we found the protein of RGN up-regulated by proteomic analysis and there was a very significant increase of SOD1 mRNA expression in liver by RT-PCR in BG vs. HG, which was consistent with the previous study.

PRDX5 (spot 5) has been shown to be a cytoprotective antioxidant enzyme that inhibits endogenous or exogenous peroxide accumulation (Knoops et al., 2011). Like peroxiredoxin, PRDX5 also functions as antioxidative and cytoprotective during oxidative stress. Over expression of human PRDX5 has been shown to inhibit peroxide accumulation and reduced cell death by exogenous peroxide in multiple organelles of CHO, HT-22, and human tendon cells (Zhou et al., 2000; Yuan et al., 2004; Zitzler et al., 2004). Meanwhile, reduced expression of PRDX5 induces cell susceptibility to oxidative damage and peroxide-induced apoptosis (Avila et al., 2008). CAT (spot 7) catalyzes the decomposition of hydrogen peroxide to water and oxygen (Chelikani et al., 2004). It serves to protect cells from the toxic effects of hydrogen peroxide. ALDH2 (spot 16) which is the second enzyme of the major oxidative pathway of alcohol metabolism and it functions as a protector against oxidative stress. Here we found PRDX5, CAT and ALDH2 over expressed in liver tissue of BG group goats compared to HG goats. GSTA1 (spot 2) plays an important role in the body’s antioxidant system. GSTA1 catalyzes the conjugation of glutathione (GSH) with products of oxidative stress, which plays an important role in protection against oxidative stress injury. GSTA1 has been found as an early indicator of acute hepatic injury in mice (Liu et al., 2014). Since over expression of PRDX5 and CAT can inhibit peroxide accumulation, we can speculate that the catalysis of GSTA1 may have been suppressed in BG goats liver as compared to the HG goats. In this study, GSTA1 was down-regulated in the BG goat livers vs. HG goats liver which was consistent with the prediction. And we also use real-time PCR analysis to observe that hepatic SOD1, GSTA1, CAT, GPX1, RGN, and PDIA3 mRNA expressions were up-regulated, all of these were relatived to anti-oxidant capacities in BG goats compared to HG goats.

T-AOC is an important integrative index used to reflect the total antioxidant capacity of the body (Cao et al., 2016). Malondialdehyde (MDA) is a major lipid peroxidation product and may reflect the degree of cellular injury (Dhalla et al., 2000), the level of MDA could reflect the degree of oxidative damage. In the study, we observed that the level of MDA was significantly decreased (*P* ≤ 0.05), the T-AOC was very significantly increased by biochemical analysis in liver of BG compared to HG goats (*P* ≤ 0.01) (Figure 3). At the same time, the plasma biochemical indexes suggested the activities of enzymes (ALT, AST, and LDH) associated with liver injury were decreased compared with BG (Table 5).

In a nutshell, our data revealed that the anti-oxidant capacities were strengthened and oxidative damage were reduced in liver of goat fed high-grain with buffering agent goats as compared to the goat fed high-grain. Previous experiments on cows and goats fed high-grain has also confirmed reduction of anti-oxidant capacities in liver (Guo et al., 2013; Duanmu et al., 2016). It consistented with our results.

### High-Grain Diet With Buffering Agent May Enhance the Body’s Stress Ability and Immunity

Heat shock cognate 71 kDa protein isoform X1 (HSPA8, spot 13) also known as heat shock 70 kDa protein 8. The heat shock protein 70 (HSPA1A, spot 12), HSPA8 and Heat shock 70 kDa protein 6 isoform X2 (HSPA6, spot 14) are members of the heat shock protein 70 (HSP 70) family and chaperone proteins. HSP 70 can act to protect cells from thermal or oxidative stress (Mosser et al., 1997). In addition, HSP 70 bound extracellular and membrane are involved in binding antigens and presenting them to the immune system (Wang et al., 2006). In this study, we found both HSPA8, HSPA1A, and HSPA6 proteins are up-regulated in BG vs. HG. Furthermore, the mRNA expressions of HSP70 were also up-regulated, the changes of hepatic HSP70 could decrease the ROS and enhance the body’s stress ability and immunity.

### High-Grain Diet With Buffering Agent Would Have an Effect on Urea Cycle

CPS-I is the first rate-limiting enzyme in the urea cycle, and it catalyzes ammonia, carbon dioxide and ATP to generate carbamyl phosphate (Summar et al., 2016). So the expression of CPS-I gene and CPS-I protein can reflect the rate of urea metabolism. ASS is the rate-limiting enzyme that catalyzes the biosynthesis of arginine, it catalyzes citrulline and aspartic acid condensation, and provides the second amino for urea synthesis (Fernandes et al., 2016). The change of enzymatic activity can adjust the rate of urea synthesis. So the expression of ASS gene can reflect the rate of urea metabolism to a certain extent. In this study we found the expression of ASS, CPS-I gene and CPS-I protein down-regulated by proteomic and qRT-PCR analysis in BG vs. HG, which revealed in the high grain group, urea metabolism was promoted, after adding buffering agent, the urea metabolism was restrained.

### High-Grain Diet With Buffering Agent Changes Amino Acid Metabolism and Glycometabolism

Glutamate dehydrogenase (GLUD, spot 10) catalyzes the oxidative deamination of Glu to 2-oxoglutarate and free NH^4+^. It is an indicator of liver function and may cause liver inflammation when its concentration is increased. It can be considered as a sensitive marker of hepatotoxicity, and highly expressed in the hepatic mitochondria. The up-regulation of GLUD has been indicated to be related the mitochondrial dysfunction (Mcgill et al., 2012). It has been reported that when the lactating dairy goats were fed with high-grain diet, GLUD was up-regulated and caused e mitochondrial dysfunction (Duanmu et al., 2016). It is proved that when buffering agent is added into the high-grain diet, the expression of GLUD is down-regulated, therefore it is concluded that the down-regulation of GLUD may reduce mitochondrial dysfunction. Cytosol aminopeptidase (LAP3, spot 23) presumably involved in the processing and regular turnover of intracellular proteins. The up-regulation of hepatic LAP3 could contribute to proper folding of newly translated and misfolded proteins and biological processes in liver of lactating dairy goats fed high-grain diet with buffering agent. The results of GO annotation analysis showed 16% proteins were classified into protein folding and 7% proteins were classified into protein refolding. Ornithine aminotransferase (OAT, spot 27) involved in the ultimate formation of the non-essential amino acid proline from the amino acid ornithine (Sharma and Dietz, 2006). The changing of OAT’s expression reveals that the metabolism between ornithine and proline was changed. The comparison of free amino acid content in hepatic vein and venous blood showed that after adding the buffering agent through HPLC, the metabolism of amino acids in the liver was changed and most of the amino acids were increasingly synthesized and decreasingly consumed in the liver.

The up-regulation of Alpha-enolase isoform X3 (ENO1, spot 21) promotes glycolysis, because ENO1 is a metalloenzyme and is responsible for the catalysis of the conversion of 2-phosphoglycerate (2-PG) to phosphoenolpyruvate (PEP), the ninth and ultimate step of glycolysis. Sorbitol dehydrogenase (SORD, spot 35) is an enzyme in carbohydrate metabolism converting sorbitol into fructose (Wu et al., 2015). With a reduced expression of SORD, the conversion amount to fructose will be reduced. Malate dehydrogenase (MDH1, spot 19) is an enzyme that reversibly catalyzes the oxidation of malate to oxaloacetate. Oxaloacetate can be converted to phosphoenolpyruvate (PEP) by phosphoenolpyruvate carboxykinase (PEPCK), thus malate dehydrogenase appears to be involved in gluconeogenesis, and it is speculated that decreased expression of MDH1 will result suppressed gluconeogenesis. It can be found that the consumption of glucose and total protein were reduced, and the synthesis of those were increased in buffering agent group by comparing the hepatic vein and portal vein plasma through HPLC.

Feeding of high-grain diet with buffering agent to lactating dairy goats, thus will cause changes in liver glycometabolism increased glycolysis and suppressed gluconeogenesis. We also find some proteins involved in carbon metabolism and biosynthesis of amino acids by KEGG pathway enrichment analysis.

## Conclusion

These results suggest that feeding of high-grain diet with buffering agent can strengthen anti-oxidant capacity, stress ability, slow down urea metabolism, alter amino acid metabolism as well as glycometabolism in the liver.

## Data Availability

All datasets generated for this study are included in the manuscript and/or the supplementary files.

## Ethics Statement

All animal procedures were approved by the Institutional Animal Care and Use Committee of Nanjing Agricultural University. The protocols were reviewed and approved, and the project number 2011CB100802 was assigned. The slaughter and sampling procedures strictly followed the ‘Guidelines on Ethical Treatment of Experimental Animals’ (2006) no. 398 established by the Ministry of Science and Technology, China and the ‘Regulation regarding the Management and Treatment of Experimental Animals’ (2008) no. 45 set by the Jiangsu Provincial People’s Government.

## Author Contributions

YZ and MH conceived and designed the experiments. MH and LL performed the experiments and analyzed the data. MH, LL, HW, and SY contributed reagents, materials, and analysis tools. MH wrote the manuscript.

## Conflict of Interest Statement

The authors declare that the research was conducted in the absence of any commercial or financial relationships that could be construed as a potential conflict of interest.

## Footnotes

1 http://geneontology.org/

## References

[B1] AlZahal O. Kebreab E. France J. McBride B. W. (2007). A mathematical approach to predicting biological values from ruminal pH measurements. *J. Dairy Sci.* 90 3777–3785. 10.3168/jds.2006-534 17638989

[B2] Annison E. F. Bryden W. L. (1998). Perspectives on ruminant nutrition and metabolism. *Nutr. Res. Rev.* 11 173–198. 10.1079/nrr19980014 19094246

[B3] Avila P. C. Kropotov A. V. Krutilina R. Krasnodembskay A. Tomilin N. V. Serikov V. B. (2008). Peroxiredoxin V contributes to antioxidant defense of lung epithelial cells. *Lung* 186 103–114. 10.1007/s00408-007-9066-2 18219526

[B4] Cao W. Xiao L. Liu G. Fang T. Wu X. Jia G. (2016). Dietary arginine and N-carbamylglutamate supplementation enhances the antioxidant statuses of the liver and plasma against oxidative stress in rats. *Food Funct.* 7 2303–2311. 10.1039/c5fo01194a 27109002

[B5] Chelikani P. Fita I. Loewen P. C. (2004). Diversity of structures and properties among catalases. *Cell. Mol. Life Sci.* 61 192–208. 10.1007/s00018-003-3206-5 14745498 PMC11138816

[B6] Dai H. Liu X. Yan J. Aabdin Z. U. Bilal M. S. Shen X. (2017). Sodium butyrate ameliorates high-concentrate diet-induced inflammation in the rumen epithelium of dairy goats. *J. Agric. Food Chem.* 65 596–604. 10.1021/acs.jafc.6b04447 28032994

[B7] Dephoure N. Gygi S. P. (2012). Hyperplexing: a method for higher-order multiplexed quantitative proteomics provides a map of the dynamic response to rapamycin in yeast. *Sci. Signal.* 5:rs2. 10.1126/scisignal.2002548 22457332 PMC5292868

[B8] Dhalla N. S. Elmoselhi A. B. Hata T. Makino N. (2000). Status of myocardial antioxidants in ischemia–reperfusion injury. *Cardiovasc. Res.* 47 446–456. 10.1016/s0008-6363(00)00078-x 10963718

[B9] Dong H. Wang S. Jia Y. Ni Y. Zhang Y. Zhuang S. (2013). Long-term effects of Subacute Ruminal Acidosis (SARA) on milk quality and hepatic gene expression in lactating goats fed a high-concentrate diet. *PLoS One* 8:e82850. 10.1371/journal.pone.0082850 24376594 PMC3871605

[B10] Duanmu Y. Cong R. Tao S. Tian J. Dong H. Zhang Y. (2016). Comparative proteomic analysis of the effects of high-concentrate diet on the hepatic metabolism and inflammatory response in lactating dairy goats. *J. Anim. Sci. Biotechnol.* 7:5. 10.1186/s40104-016-0065-0 26855776 PMC4744397

[B11] Fernandes H. S. Teixeira C. S. S. Fernandes P. A. Ramos M. J. Cerqueira N. M. (2016). Amino acid deprivation using enzymes as a targeted therapy for cancer and viral infections. *Expert Opin. Ther. Pat.* 27 283–297. 10.1080/13543776.2017.1254194 27813440

[B12] Fischer L. R. Li Y. Asress S. A. Jones D. P. Glass J. D. (2012). Absence of SOD1 leads to oxidative stress in peripheral nerve and causes a progressive distal motor axonopathy. *Exp. Neurol.* 233 163–171. 10.1016/j.expneurol.2011.09.020 21963651 PMC4068963

[B13] Guo Y. Xu X. Zou Y. Yang Z. Li S. Cao Z. (2013). Changes in feed intake, nutrient digestion, plasma metabolites, and oxidative stress parameters in dairy cows with subacute ruminal acidosis and its regulation with pelleted beet pulp. *J. Anim. Sci. Biotechnol.* 4:31. 10.1186/2049-1891-4-31 23947764 PMC3765726

[B14] Han N. Li L. Peng M. Ma H. (2016). (-)-Hydroxycitric acid nourishes protein synthesis via altering metabolic directions of amino acids in male rats. *Phytother. Res.* 30 1316–1329. 10.1002/ptr.5630 27145492

[B15] Hook S. E. Steele M. A. Northwood K. S. Wright A. D. McBride B. W. (2011). Impact of high-concentrate feeding and low ruminal pH on methanogens and protozoa in the rumen of dairy cows. *Microb. Ecol.* 62 94–105. 10.1007/s00248-011-9881-0 21625972

[B16] Ichikawa E. Yamaguchi M. (2004). Regucalcin increases superoxide dismutase activity in the heart cytosol of normal and regucalcin transgenic rats. *Int. J. Mol. Med.* 14 691–696. 15375603

[B17] Jiang X. Zeng T. Zhang S. (2013). Comparative proteomic and bioinformatic analysis of the effects of a high-grain diet on the hepatic metabolism in lactating dairy goats. *PLoS One* 8:e80698. 10.1371/journal.pone.0080698 24260456 PMC3834288

[B18] Kalyanaraman B. (2013). Teaching the basics of redox biology to medical and graduate students: oxidants, antioxidants and disease mechanisms. *Redox Biol.* 1 244–257. 10.1016/j.redox.2013.01.014 24024158 PMC3757692

[B19] Knoops B. Goemaere J. Van der Eecken V. Declercq J. P. (2011). Peroxiredoxin 5: structure, mechanism, and function of the mammalian atypical 2-Cys peroxiredoxin. *Antioxid. Redox Signal.* 15 817–829. 10.1089/ars.2010.3584 20977338

[B20] Liu F. Lin Y. Li Z. Ma X. Han Q. Liu Y. (2014). Glutathione S-transferase A1 (GSTA1) release, an early indicator of acute hepatic injury in mice. *Food Chem. Toxicol.* 71 225–230. 10.1016/j.fct.2014.06.011 24964013

[B21] Mcgill M. R. Sharpe M. R. Williams C. D. Taha M. Curry S. C. Jaeschke H. (2012). The mechanism underlying acetaminophen- induced hepatotoxicity in humans and mice involves mitochondrial damage and nuclear DNA fragmentation. *J. Clin. Investig.* 122 1574–1583. 10.1172/JCI59755 22378043 PMC3314460

[B22] Mosser D. D. Caron A. W. Bourget L. Denislarose C. Hp C. (1997). Role of the human heat shock protein hsp70 in protection against stress-induced apoptosis. *Mol. Cell. Biol.* 17 5317–5327. 10.1128/mcb.17.9.5317 9271409 PMC232382

[B23] Parent R. Beretta L. (2005). Proteomics in the study of liver pathology. *J. Hepatol.* 43 177–183. 10.1016/j.jhep.2005.04.001 15894398

[B24] Sander E. G. Warner R. G. Harrison H. N. Loosli J. K. (1959). The stimulatory effect of sodium butyrate and sodium propionate on the development of rumen mucosa in the young calf. *J. Dairy Sci.* 42 1600–1605. 10.3168/jds.S0022-0302(59)90772-6

[B25] Sharma S. S. Dietz K. (2006). The significance of amino acids and amino acid-derived molecules in plant responses and adaptation to heavy metal stress. *J. Exp. Bot.* 57 711–726. 10.1093/jxb/erj073 16473893

[B26] Shen F. Niu X. Yang D. Ying Y. Li B. Zhu G. (2010). Determination of amino acids in Chinese rice wine by Fourier transform near-infrared spectroscopy. *J. Agric. Food Chem.* 58 9809–9816. 10.1021/jf1017912 20707307

[B27] Snyder T. J. Rogers J. A. Muller L. D. (1983). Effects of 1.2% sodium bicarbonate with two ratios of corn silage: grain on milk production, rumen fermentation, and nutrient digestion by lactating dairy cows. *J. Dairy Sci.* 66 1290–1297. 10.3168/jds.s0022-0302(83)81937-7 6309932

[B28] Summar M. L. Christman B. W. Barr F. E. (2016). Therapeutic methods employing nitric oxide precursors. U.S. Patent No 9,486,429.

[B29] Ura B. Scrimin F. Arrigoni G. Franchin C. Monasta L. Ricci G. (2016). A proteomic approach for the identification of up-regulated proteins involved in the metabolic process of the leiomyoma. *Int. J. Mol. Sci.* 17:540. 10.3390/ijms17040540 27070597 PMC4848996

[B30] Wang R. Kovalchin J. T. Muhlenkamp P. (2006). Exogenous heat shock protein 70 binds macrophage lipid raft microdomain and stimulates phagocytosis, processing, and MHC-II presentation of antigens. *Blood* 107 1636–1642. 10.1182/blood-2005-06-2559 16263790

[B31] Wang X. Zhang A. Han Y. Wang P. Sun H. Song G. (2012). Urine metabolomics analysis for biomarker discovery and detection of jaundice syndrome in patients with liver disease. *Mol. Cell. Proteomics* 11 370–380. 10.1074/mcp.M111.016006 22505723 PMC3412968

[B32] Wu T. Lin D. Chen J. (2015). Changes of sorbitol content and related enzymes during fruit development in two loquat cultivars. *Actahorticulturae* 11 35–38.

[B33] Yamaguchi M. (2013). The anti-apoptotic effect of regucalcin is mediated through multisignaling pathways. *Apoptosis* 18 1145–1153. 10.1007/s10495-013-0859-x 23670020 PMC3775152

[B34] Yang Y. Tao J. Zhang Y. (2007). Preparation of protein samples from mammary tissues and its 2-DE analysis in dairy cow. *Chinese J. Animal Veterinary Sci.* 38 846–850.

[B35] Yuan J. Murrell G. A. Trickett A. Landtmeters M. Knoops B. Wang M. (2004). Overexpression of antioxidant enzyme peroxiredoxin 5 protects human tendon cells against apoptosis and loss of cellular function during oxidative stress. *Biochim. Biophys. Acta* 1693 37–45. 10.1016/j.bbamcr.2004.04.006 15276323

[B36] Zhou Y. Kok K. H. Chun A. C. Wong C. Wu H. W. Lin M. C. (2000). Mouse peroxiredoxin V is a thioredoxin peroxidase that inhibits p53-induced apoptosis. *Biochem. Biophys. Res. Commun.* 268 921–927. 10.1006/bbrc.2000.2231 10679306

[B37] Zitzler J. Link D. Schäfer R. (2004). High-throughput functional genomics identifies genes that ameliorate toxicity due to oxidative stress in neuronal HT-22 cells GFPT2 protects cells against peroxide. *Mol. Cell. Proteomics* 3 834–840. 10.1074/mcp.m400054-mcp200 15181156

